# Caveolin-1 regulates extracellular vesicle-miRNA packaging

**DOI:** 10.18632/aging.102370

**Published:** 2019-10-24

**Authors:** Heedoo Lee, Jonathan M. Carnino, Yang Jin

**Affiliations:** 1 Division of Pulmonary and Critical Care Medicine, Department of Medicine, Boston University Medical Campus, Boston, MA 02118, USA

**Keywords:** extracellular vesicle (EV), exosome, microvesicle, apoptotic body (AB),, microRNA

Keywords: **extracellular vesicle (EV), exosome, microvesicle, apoptotic body (AB), microRNA**

## 

Accumulating evidence suggests that extracellular vesicles (EVs) play an essential role in intercellular cross-talk and regulate the pathogenesis of various human diseases [1,2]. EVs are nano-sized vesicles surrounded by lipid bilayers and are generated from almost all cell types [1]. EVs facilitate intercellular communications via surface molecule-triggered signaling or by transferring EV cargo, such as proteins and RNAs, into recipient cells [1,2].

Recently, efforts have focused on studying EV-containing microRNAs (EV-miRNAs) which demonstrate the ability to carry out numerous biological functions [3]. Secretion of EVs from “parent” cells facilitates a rapid removal of intracellular miRNAs via EV packaging and release. For example, in response to gram-negative bacterial infection or LPS stimulation, macrophages actively secrete selected miRNAs, such as miR-223/142, via EV-mediated manner [4]. In quiescent macrophages, miR-223/142 inhibits NLRP3 inflammasome activation. Upon exposure to G-bacteria or LPS, miR-223/142 is uridylated and encapsulated into EVs, subsequently being released from macrophages. Due to removal of miR-223/142-mediated inhibition, macrophages are activated towards M1 polarization. This activation is reflected by enhanced phagocytosis/bacterial clearance, NO/ROS generation, and secretion of inflammatory cytokines.

EV-miRNAs potentially serve as signal transmitters among cells and facilitate cell-cell cross talk. Reports have shown that EV-cargo miRNAs can be taken up by targeted recipient cells and subsequently modulate the recipients’ intracellular events [3]. For example, lung epithelial cell-derived EV-miRNAs are taken up by lung macrophages after non-infectious stimuli. A pro-inflammatory miRNA repertoire has been identified in EVs released by epithelial cells [2,5]. After uptake by recipient macrophages, the intracellular levels of this miRNA repertoire is elevated and likely plays a role in modulating the activation of these macrophages [2,5]. Exogenous EV-cargo miRNAs likely provides an immediate way to increase intracellular levels of specific miRNA repertoires. Despite these findings, many questions remain unanswered. For instance, the quantity of EVs and EV-miRNAs that are required to be taken up by the recipient cells in order to have biological effects remains unknown. More importantly, how does the “parent” cell secrete a specific miRNA repertoire into EVs?

It is well documented that cells can secrete different miRNA profiles into EVs in response to varying stimuli or disease processes [1–3,5]. This feature has been proposed to be useful in the development of biomarkers for a variety of human diseases [1–3,5]. Recently, we attempted to delineate the underlying mechanisms by which cells secrete specific EV-miRNAs in response to noxious stimuli.

Previously, hnRNPA2B1 has been reported to bind selective miRNAs in response to stimuli and facilitate the transportation of these miRNAs into EVs [6]. After sumoylation, hnRNPA2B1 recognizes specific short motifs of miRNAs and subsequently guides these miRNAs to EVs [6]. These hnRNPA2B1-recognizable short motifs are conserved and commonly present in specific secondary structures (RNA tetraloops). Despite awareness of hnRNPA2B1’s participation in transporting miRNAs into EVs, how hnRNPA2B1 itself is transported into EVs remains unclear. Our recent work explored the detailed mechanisms by which hnRNPA2B1 is encapsulated into EVs, particularly into microvesicles (MVs). MVs are a group of EVs ranging in size from 200 nm to 1000 nm and are generated from direct budding of the cell plasma membrane [2]. Despite that the smallest category of EVs, exosomes (size less than 100 nm), may not carry a significant amount of miRNA cargo [7], we identified that MVs are a group of EVs which are miRNA rich [5]. Given that MVs are often formed from direct plasma membrane budding, we decided to examine the role of lipid raft protein caveolin-1 (cav-1) in MV formation and cargo encapsulation.

Caveolae are 50 nm to 100 nm omega-shaped invaginations of the cell surface plasma membrane and facilitate endocytosis and exocytosis of particles [8]. Cav-1, a 21- to 24-kDa protein, is the major resident scaffolding protein required to form caveolae [2,8]. We first examined whether cav-1 plays a role in the biogenesis of EVs, particularly MVs. In cav-1 null cells, MV generation is significantly reduced [2]. Over-expression of cav-1 promotes MV release from cells [2]. Thus, cav-1 may be a key regulator of MV generation and secretion from its parent cell. Interestingly, in response to oxidative stress, cav-1 and hnRNPA2B1 co-localize, interact, and transport together into secreted MVs. We used structural analysis program iterative threading assembly refinement (I-TASSER) to predict the three-dimensional (3D) binding structure of cav-1 with hnRNPA2B1. Cav-1 CSD domain and the RGG domain of hnRNPA2B1 are recognized as vital domains for the interaction. Y97 and F99 in cav-1 CSD are highly conserved sites identified to interact with hnRNPA2B1. Mutation of cav-1 Y97 and F99 suppresses interaction between cav-1 and hnRNPA2B1, as well as the MV-mediated release of hnRNPA2B1.

**Figure 1:**
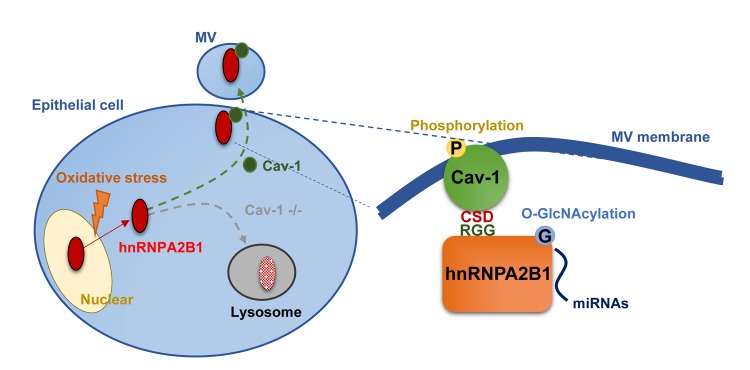
**Cav-1 is required for packaging hnRNPA2B1-bound miRNA into MVs**. Oxidative stress triggers the exportation of hnRNPA2B1, followed by the formation of cav-1/hnRNPA2B1 complex via cav-1 CSD to hnRNPA2B1 RGG domain. Cav-1 phosphorylation and hnRNPA2B1 O-GlcNacylation are responsible for sorting selected miRNAs into MVs. In the absence of cav-1, exported hnRNPA2B1 preferentially goes to the lysosomal degradation pathway.

Consistent with previous reports, we found that post-translational modifications (PTMs), specifically cav-1-Y-14 phosphorylation and hnRNPA2B1 O-GlcNAcylation, are important in the process of hnRNPA2B1-cav-1-complex-mediated miRNA secretion into MVs after oxidative stress. PTMs are now considered a fundamental mechanism by which cells control diverse biological activities (Figure 1).

In summary, stimulator-induced cav-1 and hnRNPA2B1 modifications lead to formation of cav-1/hnRNPA2B1 complex, and the cav-1/hnRNPA2B1 complex facilitates transportation of miRNAs into EVs. Interestingly, an increasing number of reports have demonstrated that caveolin-1 regulates cellular senescence and plays a role in age-related disease in vivo. In the future, one potential direction is to explore aging-related cav-1 PTM and its role in regulating EV-miRNA release.
